# Pre-Exposure to Ozone Predisposes Oak Leaves to Attacks by *Diplodia corticola* and *Biscogniauxia mediterranea*

**DOI:** 10.1100/tsw.2007.22

**Published:** 2007-03-21

**Authors:** Elena Paoletti, Naldo Anselmi, Antonio Franceschini

**Affiliations:** ^1^ IPP-CNR, Via Madonna del Piano 10, I-50019 Sesto Fiorentino, Firenze, Italy; ^2^ Dip. Protezione Piante, Univ. Tuscia, Via de Lellis, I-01100, Viterbo, Italy; ^3^ Dip. Protezione Piante, Univ. Sassari, Via de Nicola, I-07100, Sassari, Italy

**Keywords:** ozone, weak pathogen, oak, Quercus suber, Quercus cerris, Mediterranean ecosystems

## Abstract

One-year-old cork oak (*Quercus suber*) and turkey oak (*Q. cerris*) seedlings were exposed to ozone (110 ppb, 5 h day^˗1^, for 30 days) and were inoculated with Diplodia corticola and Biscogniauxia mediterranea, respectively, by spraying a suspension of spores on the leaves. Both fungi are endophytic and may act as weak parasites, contributing to oak decline. Ozone exposure stimulated leaf attacks after inoculation, although the physiological, visible, and structural responses of both oaks to O_3_ exposure were weak. In fact, steady-state gas exchange, leaf waxes, and wettability were not significantly affected by O_3_. In *Q. cerris*, O_3_ altered the structure of stomata, as observed by scanning microscopy, and reduced the leaf relative water content. No hyphal entry through stomata or growth towards stomata was, however, observed. Inoculations were performed in a humid chamber at low light; stomata were likely to be closed. When *Q. cerris* was inoculated in natural conditions, i.e., in a forest infected by *B. mediterranea*, seedlings pre-exposed to the enhanced O_3_ regime had a higher number of *B. mediterranea* isolates than the controls. This suggests that pre-exposure to O_3_ predisposed *Q. cerris* leaves to attacks by *B. mediterranea* independent of stomata. The hyphae of both fungi were able to enter the leaf through the cuticle, either by gradual in-growth into the cuticle or erosion of a hollow in the cuticle at the point of contact. The primary cause of increased leaf injury in O_3_-exposed seedlings appeared to be higher germination of spores than on control leaves.

## INTRODUCTION

Forest decline results from a combination of interacting factors[1]. The terminal stage of decline often involves pathogens or pests that move from a latent to an active phase in trees under stress. Ozone ($O_3$) is a pervasive air pollutant in most parts of the world[2]. Pathogens are believed to colonize plants weakened by $O_3$[3]. There are extensive reports of the effects of $O_3$ on trees, but few reports link $O_3$ exposure with tree diseases[3].

Several factors including drought, severe summer flooding, changes in agronomic practices, wood-boring insects, and fungal diseases[4] have been implicated in oak decline in Southern Europe. The role of $O_3$ pollution is unknown. Both *Biscogniauxia mediterranea* and *Diplodia corticola* are endophytic fungi that can affect plants of different ages, inducing bark cankers or symptoms similar to those produced by a tracheomycotic disease, respectively. *Diplodia corticola* (formerly *D. mutila*) is considered to be one of the main causes of *Quercus suber* decline[5]. *Biscogniauxia mediterranea* (formerly *Hypoxylon mediterraneum)* has been frequently associated with oak decline in the Mediterranean area[6], mostly affecting *Q. cerris*[7,8].

Our objective was to clarify whether $O_3$-exposed leaves of two Mediterranean oaks, *Q. suber* and *Q. cerris*, were more susceptible to *D. corticola* and *B. mediterranea*, respectively, and to test some $O_3$ responses (amount of epicuticular waxes, leaf wettability, stomata structure, gas exchange, leaf water content) that may affect leaf susceptibility to fungi.

## MATERIALS AND METHODS

### Plant Material and Ozone Exposure

One-year-old, nursery-grown, potted, uniform-sized seedlings of *Q. suber* and *Q. cerris* were placed in controlled conditions (20–23°C, 70–80% RH, 500 µmol $m^{-2}$ $sec^{-1}$ PAR, 12-h photoperiod, charcoal-filtered air) for 1 week at the end of July 2001. On August 1st, seedlings were placed in exposure chambers, either in charcoal-filtered air, or to elevated $O_3$. The elevated $O_3$ exposure averaged 110 ppb (in the form of a square wave, 5 h $day^{-1}$) for 30 days, corresponding to 10.5 ppm h AOT40. The $O_3$ exposure was designed to be typical of peak exposures in Italy[9]. Enclosures were supplied with charcoal-filtered air and a complete air exchange was achieved every 1.5 min. A fan mixed the internal air. Ozone was generated with a Helios Italquartz generator (Model Heliozon, Milan, Italy), metered to the chambers with mass flowmeters (Model GFC171, Aalborg, NY), and monitored with an $O_3$ monitor (Model 202, 2B Technologies, Boulder, CO). Plants were watered to field capacity once a week.

### Preinoculation Measurements

Gas exchange, epicuticular waxes, leaf wettability, and water content were measured shortly after the end of $O_3$ exposure (August 30th) and before fungal inoculation (August 31st, afternoon) on three seedlings per species and treatment. The low replication was chosen to perform all the measurements at 12:00–13:00 (gas exchange) and to reduce destructive sampling and wounding that might have favored hyphae entering the leaf (epicuticular waxes, leaf wettability, and water content). Steady-state net photosynthesis and stomatal conductance were assessed on three leaves per seedling by IRGA (CIRAS-1, PP-Systems, Herts, U.K.) that controlled temperature (25°C), leaf-to-air vapor pressure difference (2.0 ± 0.2 kPa), light (1400 µmol $m^{-2}$ $sec^{-1}$ PAR), and $CO_2$ concentration (365 ppm). To extract epicuticular waxes, 1 g of fresh leaves per seedling was shaken for 10 sec with 50 ml chloroform[10]. The solution was filtered through a 0.2-µm PTFE membrane in a preweighed aluminum container, and allowed to evaporate to constant weight in a fume cupboard at room temperature. The residue weight was cross-referenced to leaf area determined with a leaf area meter AM 300 (ADC, Herts, U.K.). Wettability of the adaxial leaf surface was assessed as drop contact angle (DCA) using a bench microscope with a protractor graticule[11]. A 2-$cm^2$ stripe was cut in the central part of each fresh leaf (one per seedling), halfway between the midvein and the margin. A 1-µl distilled water droplet was put to each leaf stripe using a 5-µl syringe, and contact angle (±1°) was immediately measured. As the sparse pubescence on the adaxial leaf surface of *Q. suber* induced erratic values, DCA was measured in *Q. cerris* only. Leaf water content was measured only in *Q. cerris*. Five 1-$cm^2$ leaf discs per seedling were sampled, avoiding the midrib. Relative water content was calculated as $RWC = 100*[(FW – DW)/(SW – DW)]$ where FW, DW, and SW are fresh, dry, and saturation weight, respectively. SW was measured after one night in Petri dishes with wet blotting paper. DW was measured after oven drying at 80°C until reaching a constant weight.

## Inoculation in Controlled Conditions

Mature *B. mediterranea* stromata on cut *Q. cerris* branches were soaked with sterile deionized water and put in a humid chamber at 100% RH (measured with a hygrothermograph). At sporulation, ascospores were collected in sterile containers and combined in a single sprayer containing 1 l sterile water. The number of spores, determined with a cell-count slide, was $10^6$ ascospores $ml^{-1}$, i.e., the density that stimulates the best *in vitro* germination rate[7]. For inducing picnidia differentiation in *D. corticola*, *Q. suber* leaves were sterilized and put in Petri dishes containing PDA medium infected with *D. corticola* mycelium, at ambient light and temperature. After 30 days, picnidia were moved to sterile vials with 200 μl of sterile water. They were crushed and shaken to favor conidia dissemination. The conidia suspension obtained was adjusted at $10^5$ conidia $ml^{-1}$.

On August 31st, six control and six $O_3$-exposed seedlings of each species were sprayed with the suspensions of *B. mediterranea* ascospores (*Q. cerris*) or *D. corticola* conidia (*Q. suber*) until upper and lower leaf blades were fully wet. Inoculated seedlings were immediately sealed in one humid chamber per pathosystem (100 × 50 × 100 cm, 100% RH, 200 μmol PAR $m^{-2}$ $sec^{-1}$) at 20 or 25°C, for *Q. cerris* or *Q. suber*, respectively. The temperatures were set at the optimum for germination of B. mediterranea[7] and D. corticola[12] spores, respectively. After a 2-day incubation, macroscopical and microscopical injury was observed.

## Visible Injury

No visible injury was observed on $O_3$-exposed leaves. Inoculations caused leaves to turn brownish-black, starting from edges to the midrib. The extent of such necroses was determined on ten leaf sections for each seedling with the WinDias image analysis system (Delta-T Devices, Cambridge, U.K.). Observations were carried out immediately after the 2-day incubation on September 2nd. For *Q. suber*, additional observations were carried out at day 2 and 5 after incubation, on September 4th and 7th, respectively.

## Microscopic Observations

After incubation, three injured leaves per plant were collected. To observe the adaxial and abaxial leaf surfaces, two 0.25-$cm^2$ squares were cut in each of the brownish and green part of the leaf, immediately coated with gold (1.5 min, Edwards S150A Sputter Coater), and observed using a scanning electron microscope (SEM 505 Philips, Eindhoven, Holland) at 5 kV. The low voltage helped to reduce damage to the fresh material. Drying or use of acetone would have altered the fungal structures. Fifty stomata per leaf portion were assigned to one of four damage classes: 0, no injury; 1, slight injury; 2, medium injury; 3, severe injury. The structural damage to stomata was quantified as SDI (Stomatal Damage Index) = $[(N1 * 1) + (N2 * 2) + (N3*3)]/(N0 + N1 + N2 + N3)$, where N was the number of stomata in each damage class[13]. The abaxial surface of *Q. suber* leaves was covered by a dense pubescence, and so damage to stomata was not assessed. Ascospores, conidia, and hyphae were also observed. Quantification was impractical because of the patchy nature of infection.

## Inoculation in Natural Conditions

After $O_3$ exposure (August 31st), 30 additional *Q. cerris* seedlings (15 control and 15 $O_3$-exposed seedlings) were placed in a *Q. cerris* forest in decline because of *B. mediterranea*. September was dry, but October precipitation totaled 130 mm, which promoted natural fungal inoculation of the seedlings. Leaves on the seedlings were already senescent. On November 1st, fragments of outer sapwood and buds were removed from the seedlings with a sterile knife, placed in Petri dishes containing Potato Dextrose Agar, and incubated at 23°C in the dark. Fungal isolates were identified morphologically or by molecular methods, as described in Mazzaglia et al.[14].

## RESULTS

Ozone exposure did not induce leaf visible injury. The $O_3$-induced reduction in net photosynthesis and stomatal conductance, as well as in the amount of epicuticular waxes and drop contact angle, was not statistically significant (Table 1). In contrast, $O_3$ exposure significantly decreased and increased, respectively, leaf relative water content and SDI of *Q. cerris* (Table 1), which was the only species that allowed the determination of these parameters.

**TABLE 1**
**Parameters Measured in Control (Charcoal-Filtered) and $O_3$-Exposed (110 ppb $O_3$, 5 h day$^{-1}$, 30 days, 10.5 ppm h AOT40) Leaves of *Q. cerris* and *Q. suber****

| Parameter | *Q. cerris* | | *Q. suber* | |
|---|---|---|---|---|
| | Control | $O_3$-Exposed | Control | $O_3$-Exposed |
| Net photosynthesis (μmol m$^{-2}$ sec$^{-1}$) | 9.27 a (3.82) | 5.88 a (2.64) | 10.36 a (2.29) | 8.54 a (3.47) |
| Stomatal conductance (mmol m$^{-2}$ sec$^{-1}$) | 94 a (51) | 58 a (18) | 235 a (57) | 227 a (70) |
| Relative water content (%) | 82 a (3) | 74 b (2) | — | — |
| Epicuticular wax (μg cm$^{-2}$) | 26 a (4) | 20 a (4) | 30 a (5) | 26 a (5) |
| DCA (°) | 104 a (3) | 99 a (2) | — | — |
| SDI | 0.10 b (0.01) | 0.26 a (0.10) | — | — |

* Standard deviation in parenthesis (n = 3). Different letters show significant differences between control and $O_3$-exposed leaves for each species.

Ozone-exposed leaves exhibited more severe visible injury than control leaves in the *Q. cerris – B. mediterranea* pathosystem after a 2-day incubation period in a humid chamber, and in the *Q. suber – D. corticola* pathosystem at both day 2 and 5 (Fig. 1). When *Q. cerris* inoculation was carried out in a forest infected by *B. mediterranea*, the percentage of fungal isolates that were *B. mediterranea* was higher in previously $O_3$-exposed seedlings than in controls (Fig. 2).

Scanning microscopy observations indicated many spores on both control and $O_3$-exposed leaves at the end of the 2-day incubation. However, most spores on control leaves did not germinate (Fig. 3A). Most hyphae were on the symptomatic leaf parts, on both the adaxial and abaxial blade. Hyphae were never observed to enter into stomata (Figs. 3B and 4C) and did not appear to grow towards stomata. However, observation of *Q. suber* stomata was limited by the trichome network covering the abaxial blade (Fig. 4C). Hyphae entered the leaf through the cuticle, embedding into the epicuticular waxes (Figs. 3C and 4A), and sometimes eroding hollows (Figs. 3C and 4B). *D. corticola* hyphae showed a right-angle bend at the penetration point (Fig. 4A). On $O_3$-exposed *Q. cerris* leaves, the anamorph of *B. mediterranea* was observed (Fig. 3D).

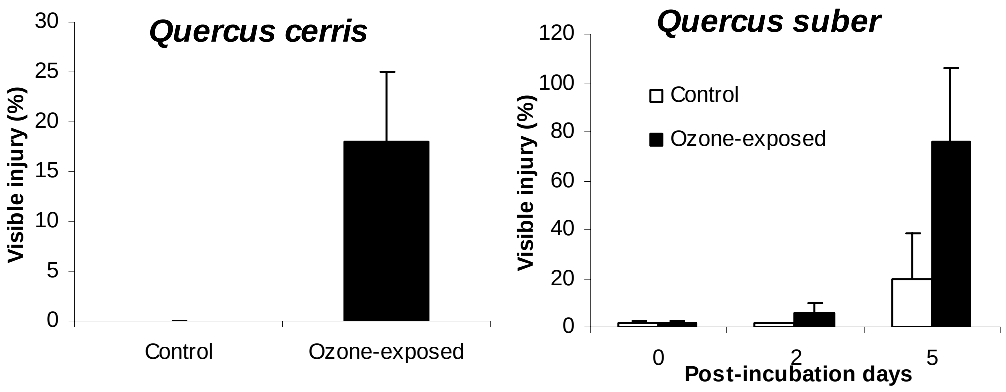

**FIGURE 1.** Percent of injured vs. total leaf area (+SD) after a 2-day incubation (day 0) of control (charcoal-filtered) and $O_3$-exposed (110 ppb $O_3$, 5 h $day^{-1}$, 30 days) seedlings in a humid chamber. *Quercus cerris* and *Q. suber* were inoculated with suspensions of *B. mediterranea* ascospores and *D. corticola* conidia, respectively. In *Q. suber*, leaf visible injury was assessed also at day 2 and 5 after incubation. n = 6.

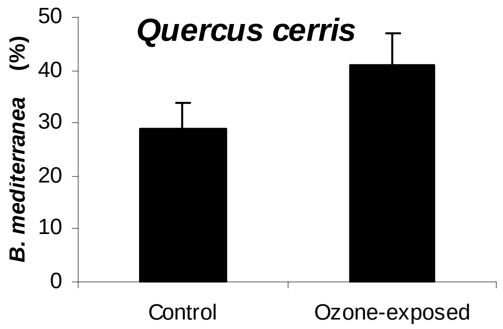

**FIGURE 2.** Percent of fungal isolates from foliage of *Q. cerris* seedlings that were *B. mediterranea* isolates (+SD) in control (charcoal-filtered) and $O_3$-exposed (110 ppb $O_3$, 5 h $day^{-1}$, 30 days) seedlings after a 1-month exposure in a *Q. cerris* forest naturally infected by *B. mediterranea*. n = 15.

## DISCUSSION

A 30-day acute $O_3$ exposure did not affect steady-state gas exchange, leaf waxes, and wettability of either oak species. Mediterranean vegetation, mostly evergreen sclerophylls like *Q. suber*, are considered more $O_3$ tolerant than mesophilic vegetation[15]. In *Q. cerris*, $O_3$ altered the structure of stomata, as observed by scanning microscopy, and reduced the leaf relative water content. Decreased relative water content is among the most sensitive plant responses to $O_3$[16]. Higher drought-induced susceptibility to *B. mediterranea*[17,18] and *D. corticola*[19,20] on *Q. cerris* and *Q.* species, respectively, has been reported relative to controls. The fast growth of *B. mediterranea* mycelium in embolized vessels was also reported in drought-stressed oaks[18].

There was a marked increase in visible injury observed in $O_3$-exposed leaves experimentally infected by *B. mediterranea* (18% vs. 0% in controls at day 0 after incubation) or *D. corticola* (+6, 267, and 280% at day 0, 2, and 5, respectively). No hyphal entry through stomata or growth attraction towards stomata (stomatal tropism, *sensu*[21]) was observed. As inoculations were performed in a humid chamber at low light, stomata were likely to be closed. When *Q. cerris* was inoculated under natural forest conditions, a higher proportional number of *B. mediterranea* isolates were observed on leaves of $O_3$-exposed seedlings. This suggests that preinoculation $O_3$ exposure increased susceptibility of *Q. cerris* leaves to *B. mediterranea*

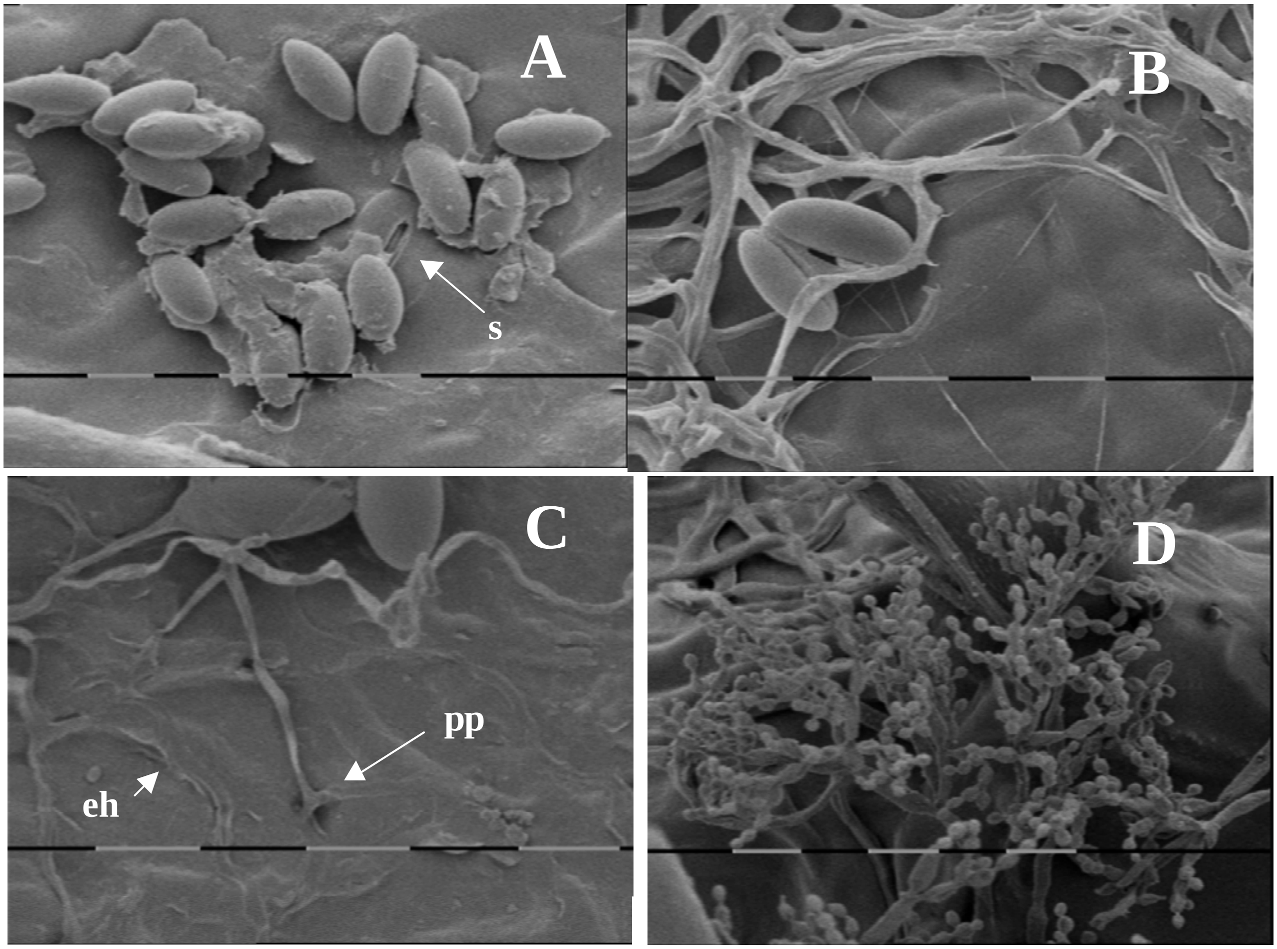

**FIGURE 3.** *Quercus cerris* leaves sprayed with a suspension of *B. mediterranea* ascospores. Control leaf: A, ungerminated spores (S = stoma) (abaxial blade). Ozone-exposed leaf (110 ppb $O_3$, 5 h $day^{-1}$, 30 days): B, germinating spores and hyphae development (abaxial blade); C, germinating spores and entry of an hypha through the cuticle (pp = penetration point; eh, hypha embedded into the wax layer) (adaxial blade); D, anamorph of *B. mediterranea* (adaxial blade). White bar = 10 μm.

attack independently of stomata. The hyphae of both fungal species were able to enter the leaf directly through the cuticle. Two ways of entry into the leaf were observed: gradual embedding into the epicuticular waxes, and erosion of a hollow in the cuticle at the point of hyphal contact. The cuticular erosion may be caused by cuticle-degrading enzymes. Host colonization is known to be facilitated by enzyme activity in both *Biscogniauxia*[22] and *Diplodia*[23] species. In *D. corticola*, entry into the mesophyll was preceded by a right-angle bend of the hypha that may be considered as an appressorium[24].

The slight $O_3$-induced reduction in the amount of epicuticular waxes and increase in leaf wettability may have favored fungal penetration of the cuticle. However, the main cause of the increased leaf injury in $O_3$-exposed seedlings appeared to be the higher germination rate of spores than in control leaves, mainly in the *Q. cerris – B. mediterranea* pathosystem. Ozone may alter the chemical composition of epicuticular waxes[25], thus changing the environment for spore attachment and germination. Ozone may alter content and quality of leaf nitrogen[26] and leaf carbohydrates[27], thus rendering the leaves a better substrate for fungi. In *Populus maximowiczii* and *P. deltoides* B-60, tolerant and sensitive to *Melampsora larici-populina*, respectively, spore germination and tube development were affected by the host leaf chemical characteristics[28]. However, $O_3$ may elicit metabolic responses similar to those induced by a pathogen attack, such as hypersensitive responses, phenols, reactive oxygen species[29], possibly inducing cross-tolerance, i.e., the opposite response of what this study reports.

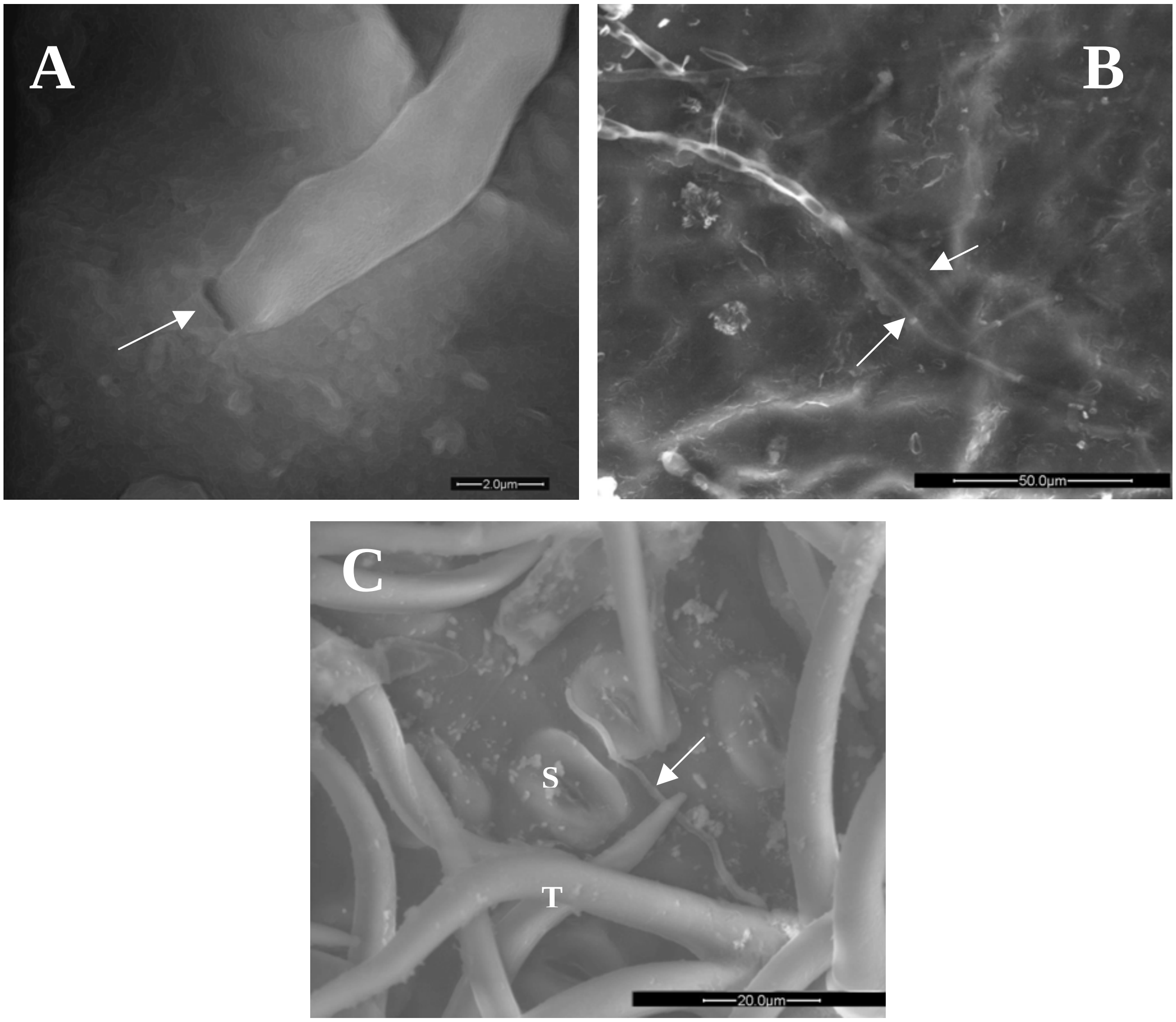

**FIGURE 4.** *Quercus suber* leaves sprayed with a suspension of *D. corticola* conidia. Control leaf: A, penetration through the cuticle by inducing a hollow (arrow) (adaxial blade); B, hyphae embedded into the wax layer (arrows) (adaxial blade). Ozone-exposed leaf (110 ppb $O_3$, 5 h $day^{-1}$, 30 days): C, development of an hypha (arrow) (S = stoma, T = trichome) (abaxial blade).

In conclusion, pre-exposure to $O_3$ increased susceptibility of *Q. suber* and *Q. cerris* leaves to successful fungal attacks by *D. corticola* and *B. mediterranea*, respectively, although mechanisms behind findings still await clarification.

Ozone and drought are the most important abiotic stress factors affecting Mediterranean forests[15]. Climatic conditions that increase $O_3$ concentrations favor drought stress as well. Drought is a predisposing factor in the pathogenesis of *B. mediterranea* on *Q. cerris*[17,18] and of *D. corticola* on *Quercus* species[19,20]. Increased attacks after $O_3$ exposure may contribute to the oak decline caused by fungal parasites in Mediterranean ecosystems.

## ACKNOWLEDGMENT

The authors are grateful to the Italian Ministry of Education, University and Research for financial support (PRIN project: Ruolo degli endofiti fungini nel deperimento delle querce); to Giacomo Lorenzini and Cristina Nali for the help in performing ozone exposures; and to Laura Bonzi for the pictures in Fig. 4.
